# The effectiveness of Traditional Chinese Medicine in treating cancer related anemia: a systematic review and meta-analysis

**DOI:** 10.3389/fmed.2026.1739128

**Published:** 2026-02-24

**Authors:** Quan-yao Li, Zi-yu Zhang, Wen-xiao Yang, Hui Liu, Xi Li, Wen-chao Qiu, Zhao-wei Guo, Jun Shi

**Affiliations:** 1 Shanghai Fourth People’s Hospital Affiliated to Tongji University, Shanghai, China; 2 Yueyang Hospital of Integrated Traditional Chinese and Western Medicine Affiliated to Shanghai University of Traditional Chinese Medicine, Shanghai, China

**Keywords:** cancer related anemia, Chinese herbal, hemoglobin, meta-analysis, quality of life

## Abstract

**Objective:**

The aim is to systematically evaluate the clinical efficacy of Traditional Chinese Medicine (TCM) in the treatment of cancer related anemia (CRA), and to provide higher quality evidence-based medical evidence for the treatment of CRA with TCM.

**Methods:**

Pubmed, Embase, Cochrane library, Scopus, Web of Science, China National Knowledge Infrastructure (CNKI), China Biology Medicine Disk (CBM) and WanFang were searched for the literature on TCM treatment of CRA included in the databases from the establishment of the databases to December 2025. Meta-analysis was performed using ReVman5.3 software.

**Results:**

6,201 relevant literatures were retrieved. According to inclusion and exclusion criteria, a total of 58 literatures were included, with a total of 4,308 patients, including 2,157 patients in the treatment group and 2,151 patients in the control group. The treatment group was better than the control group in improving hemoglobin level, clinical efficacy, KPS score, TCM syndrome efficacy, TCM Syndrome Score, immune function and red blood cell count, the difference was statistically significant (*P* < 0.05). By drawing a funnel figure of hemoglobin levels, it was found that the left and right sides of the funnel figure were basically symmetrical, indicating that the publication bias of this study was low. It can be considered that TCM treatment can significantly improve hemoglobin and improve the anemia of patients.

**Conclusion:**

Traditional Chinese Medicine can significantly improve CRA, enhance TCM syndrome and immune function, and improve patients’ quality of life.

**Clinical Trial Registration:**

https://www.chictr.org.cn, identifier [ChiCTR2300077699].

## 1 Introduction

Cancer related anemia (CRA) is a common complication occurring during the development and treatment of tumors (1). Its primary manifestations include fatigue, pallor, and dizziness. CRA has a high occurrence rate, studies have shown that the incidence of CRA in China reaches 49.24%, and the incidence rises to as high as 90% after chemotherapy. However, the treatment rate remains low, with up to 92.84% of CRA patients receiving no anemia-correcting therapy (2), leading to aggravated anemia symptoms and severely compromised quality of life. Relevant studies indicate that cancer patients with anemia have a 65% increased overall risk of mortality compared to those without anemia (1). CRA is generally induced by multiple factors, including cancer-related factors, and antitumor treatments. Current evidence tends to implicate inflammation, malnutrition, renal impairment, abnormal iron metabolism, and bone marrow hematopoietic dysfunction, among others (3). The main modern medical treatments for CRA include blood transfusion, erythropoiesis-stimulating agents (ESAs), and iron supplementation. Although blood transfusion can rapidly correct blood loss, it carries risks of complications such as infection, hemolysis, and thrombosis (4). According to the 2022 guidelines of the Chinese Society of Clinical Oncology (3), erythropoietin (EPO) therapy has certain limitations and is not recommended, and it exhibits a delayed onset of action, and is effective in only a subset of patients. Iron supplements are only about 10% absorbed by the human body and are often associated with severe gastrointestinal irritation. Traditional Chinese Medicine (TCM) has a long history in the treatment of CRA and offers unique advantages with a favorable safety profile. Therefore, it is of great significance to actively investigate the therapeutic role of TCM in CRA. Although preliminary studies (5, 6) have explored the role of TCM in the treatment of CRA, limitations such as earlier search timeframes, limited database coverage, and insufficient assessment of evidence quality remain. Therefore, this study further updates the search period, incorporates databases including Embase, Scopus, and Web of Science, strictly adheres to the PRISMA guidelines, applies the GRADE approach to grade the quality of evidence, and systematically evaluates the clinical efficacy of oral Chinese herbal medicine in treating CRA. The aim of this study is to provide higher-quality evidence-based support for TCM in the treatment of CRA through a more comprehensive, rigorous, and updated evidence synthesis.

## 2 Research methods

The study was conducted in strict compliance with the PRISMA guidelines for systematic reviews and meta-analyses.

### 2.1 Literature search

The literature search strategy was developed in accordance with the Cochrane Systematic Reviews Handbook guidelines. The following databases were searched from their inception to December 2025 for relevant clinical studies on the treatment of CRA with TCM, such as PubMed, Embase, Cochrane Library, Scopus, Web of Science, China National Knowledge Infrastructure (CNKI), China Biology Medicine Disk (CBM), and WanFang. The search terms included “Cancer related anemia,” “Tumor related anemia,” “Anemia,” “Traditional Chinese Medicine,” “Chinese herbal,” “Herbal medicine,” “TCM,” “Randomized” and “RCT.” And the search strategy was adjusted according to different databases. The search strategy takes pubmed as an example: ((((Cancer related anemia) OR (Tumor related anemia)) OR (Anemia)) AND ((((Traditional Chinese Medicine) OR (Chinese herbal)) OR (Herbal medicine)) OR (TCM))) AND ((Randomized[Title/Abstract]) OR (RCT[Title/Abstract])). In addition, we manually searched for relevant studies to obtain as much information as possible. The manual search method is as follows: (1) scanning the reference lists of all included articles and relevant systematic reviews. (2) Searching the trial registries ClinicalTrials.gov, Chinese Clinical Trial Registry, and the International Traditional Medicine Clinical Trial Registry. (3) Reviewing the tables of contents of key Chinese medicine journals (e.g., Zhongguo Za Zhi, Zhongguo Zhong Yao Za Zhi) from the past 10 years.

### 2.2 Inclusion criteria

(1) Design: randomized controlled trials (RCTs).

(2) Participant: patients with a histologically or cytologically confirmed diagnosis of malignant tumors who also met the diagnostic criteria for anemia.

(3) Interventions: the control group received conventional or symptomatic treatment (e.g., iron supplements, folic acid, EPO, etc.). The treatment group received oral Chinese herbal medicine (either proprietary Chinese medicine or decoction), either as a standalone intervention or in addition to the control group’s regimen.

(4) Outcomes: Primary Outcome: Hemoglobin level (g/L). Secondary Outcomes: Clinical efficacy, Red blood cell count, Karnofsky Performance Status (KPS) score, TCM syndrome score, TCM syndrome efficacy, and immune function indicators (CD3+, CD4+, CD8+, CD4+/CD8+).

(5) Publication: full-text articles published in peer-reviewed journals.

### 2.3 Exclusion criteria

(1) Conference literature.

(2) Literature from which valid data could not be extracted.

(3) Duplicate publications.

### 2.4 Data extraction

Two researchers independently screened the literature according to the inclusion and exclusion criteria. Initial screening was performed based on the title, abstract, and keywords, followed by a full-text review for secondary screening. The screening results were cross-checked by both researchers. Any discrepancies were resolved through consultation with a third researcher holding a senior professional title. The primary extracted information including author(s), year of publication, group size, randomization method, interventions, and outcome measures.

### 2.5 Quality assessment criteria

The Cochrane-recommended risk of bias assessment tool, RoB 2.0, was used to evaluate the quality of the included literature. This assessment covered the following domains: randomization process, deviations from intended interventions, definite outcome indicator, outcome measurement, and selection of the reported result. Two researchers independently performed the risk assessment. Any discrepancies were resolved through consultation with a third researcher.

### 2.6 Statistical analysis

Meta-analysis of the included literature was performed using RevMan software (version 5.3). Firstly, a heterogeneity testing was conducted for each study. If no statistical significance was observed (*P* > 0.05, I^2^ < 50%), the included studies were considered to lack heterogeneity, and a fixed-effects model was applied for analysis. Conversely, if statistical significance was present (*P* < 0.05, I^2^ > 50%), heterogeneity among the included studies was indicated, necessitating further investigation into its sources to determine the suitability of a random-effects model for analysis. For dichotomous data, the odds ratio (OR) with a 95% confidence interval (CI) was used as the statistical measure for efficacy analysis. For continuous data, the mean difference (MD) with a 95% CI was employed as the statistical measure for efficacy analysis.

## 3 Results

### 3.1 Literature search results

**FIGURE 1:**
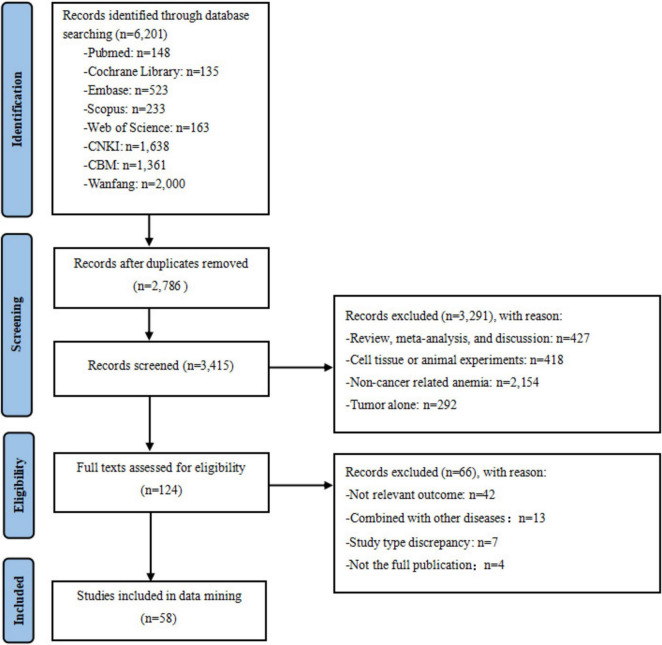
Flow chart of literature search.

This study identified 6,201 relevant articles through literature search. After removing 2,786 duplicate records using Note Express software. Subsequent screening of remaining articles through title and abstract analysis removed 3,291 articles. The full texts of the remaining 124 articles were thoroughly reviewed. Finally, according to the inclusion and exclusion criteria, 58 articles were included in the study. See Figure 1.

### 3.2 Basic characteristics and quality assessment of included literature

A total of 58 randomized controlled trials were included in this study, involving 4,308 patients with CRA. Among these patients, 2,157 were assigned to the treatment group and 2,151 to the control group. The baseline characteristics, such as age and gender, were consistent across the included studies, indicating comparability among the study groups. See Table 1.

**TABLE 1:** Basic characteristics of the included literature.

Included literature	Sample size		Grouping	Baseline	Intervention		Outcome measures
	Control group	Treatment group			Control group	Treatment group	
Bai (36)	44	44	Simple randomization	Coincide	EPO	EPO+TCM	➀➁➂➃
Cao et al. (38)	30	30	Randomization	Coincide	EPO	EPO+TCM	➀➁➂➃➄➆➉
Chen (35)	30	30	Randomization	Coincide	Symptomatic treatment	Standard chemotherapy+TCM	➀➁➂➆➇➈➉
Chen et al. (39)	44	44	Randomization	Coincide	Conventional treatment	Conventional treatment+TCM	➀➁➂➃➄➇➈➉
Cheng et al. (66)	32	33	Lottery method	Coincide	EPO	EPO+TCM	➀➁➂
Cong (7)	40	40	Random number table	Coincide	Conventional treatment	Conventional treatment+TCM	➀➁➂
Feng et al. (8)	64	64	Random number table	Coincide	Symptomatic treatment	Symptomatic treatment+TCM	➀➁➂➄
Fu et al. (9)	28	32	Random number table	Coincide	Conventional treatment	TCM	➀➁➂➃➄➇➈➉
He et al. (10)	40	40	Random number table	Coincide	Conventional treatment	Conventional treatment+TCM	➀➁➂
Hu and Jiang (40)	32	34	Randomization	Coincide	Donkey-hide gelatin granules	TCM	➀➁➂➅➆➇➈➉
Hu and Liao (33)	30	30	Envelope method	Coincide	EPO	EPO+TCM	➀➁➂➃➅
Huang (41)	22	23	Randomization	Coincide	Shengxue Ning+EPO	Shengxue Ning+EPO+TCM	➀➁
Huang et al. (11)	30	30	Random number table	Coincide	Conventional treatment	TCM	➀➂➆➇➈➉
Huang et al. (42)	30	30	Randomization	Coincide	Xiangsha LiuJun BuGuZhi Decoction combined with SiWu Decoction	Xiangsha LiuJun BuGuZhi Decoction combined with SiWu Decoction+TCM	➀➁➂➃➄
Li (67)	35	35	Lottery method	Coincide	Compound Donkey-hide gelatin slurry	TCM	➀➁➃
Li (43)	40	40	Randomization	Coincide	Symptomatic treatment	Symptomatic treatment+TCM	➀➂➃
Li (44)	60	60	Randomization	Coincide	EPO	EPO+TCM	➀➁➂
Li (12)	30	30	Random number table	Coincide	EPO	EPO+TCM	➀➁➂➃➄➅
Li and Jin (13)	30	30	Random number table	Coincide	Ferralia	Ferralia+TCM	➀➁➂
Li (14)	45	45	Random number table	Coincide	EPO	EPO+TCM	➀➁➂➃
Li et al. (45)	50	50	Randomization	Coincide	Conventional treatment	Conventional treatment+TCM	➀➁➂➄➉
Liang (46)	75	75	Randomization	Coincide	EPO	EPO+TCM	➀➁➂
Liu et al. (47)	22	22	Randomization	Coincide	Conventional treatment	Conventional treatment+TCM	➀➂
Liu et al. (15)	30	30	Random number table	Coincide	Conventional treatment	TCM	➀➁➂➅➆➇➈➉
Liu and Wang (16)	35	35	Random number table	Coincide	Symptomatic treatment	Symptomatic treatment+TCM	➀➁➂➃➄➇➈➉
Liu (17)	36	36	Random number table	Coincide	Symptomatic treatment	Symptomatic treatment+TCM	➀➁➂➄➅
Lou et al. (48)	30	30	Randomization	Coincide	Conventional treatment	Conventional treatment+TCM	➀➂➄➅
Lu et al. (18)	29	26	Random number table	Coincide	EPO	EPO+TCM	➀➁
Lu (37)	30	30	Simple randomization	Coincide	Symptomatic treatment	TCM	➀➁➄➅
Ma (19)	30	30	Random number table	Coincide	Symptomatic treatment	TCM	➀➂➃➄
Ma (49)	40	40	Randomization	Coincide	Symptomatic treatment	Symptomatic treatment+TCM	➀➂➃➄
Niu (20)	27	27	Random number table	Coincide	Conventional treatment	TCM	➀➁➂➃
Peng et al. (50)	40	40	Randomization	Coincide	Symptomatic treatment	TCM	➀➂➃➆➇➈➉
Qi and Wang (21)	24	24	Random number table	Coincide	Symptomatic treatment	TCM	➀➁
Qiao (51)	21	21	Randomization	Coincide	Symptomatic treatment	Symptomatic treatment+TCM	➀➁
Ren and Bie (22)	40	40	Random number table	Coincide	EPO	EPO+TCM	➀➁➂
She et al. (52)	29	30	Randomization	Coincide	Compound Donkey-hide gelatin slurry	TCM	➀➂
Song et al. (23)	50	50	Random number table	Coincide	Symptomatic treatment	Symptomatic treatment+TCM	➀➁➂➃
Sun et al. (53)	56	65	Randomization	Coincide	Symptomatic treatment	TCM	➀➁➂➆➇➈➉
Tuo (24)	32	32	Random number table	Coincide	EPO	TCM	➀➁➂➃➄➅➆➇➈➉
Wang (54)	30	30	Randomization	Coincide	EPO	EPO+TCM	➀➁➂
Wang and Li (25)	40	40	Random number table	Coincide	Symptomatic treatment	TCM	➀➁➂➃➆➇➈➉
Wang et al. (26)	55	55	Random number table	Coincide	Symptomatic treatment	TCM	➀➁➂➆➇➈➉
Wang and Li (55)	40	40	Randomization	Coincide	Conventional treatment	TCM	➀➁➂➄➆➇➈➉
Wang (56)	30	30	Randomization	Coincide	EPO	EPO+TCM	➀➁➂➄➅➆
Wang et al. (27)	40	40	Random number table	Coincide	EPO	TCM	➀➁➂➄
Wang (28)	35	35	Random number table	Coincide	EPO	EPO+TCM	➀➁➃
Wang (57)	38	38	Randomization	Coincide	Symptomatic treatment	Symptomatic treatment+TCM	➀➁
Wen et al. (29)	30	30	Random number table	Coincide	EPO	EPO+TCM	➀➁➂
Xu and Long (58)	34	34	Randomization	Coincide	Symptomatic treatment	Symptomatic treatment+TCM	➀➁➂➅
Yao (30)	45	45	Random number table	Coincide	EPO	EPO+TCM	➀➁➂➄➆➇➈➉
Yu et al. (34)	34	33	Envelope method	Coincide	EPO	EPO+TCM	➀➁➂➄
Yu (59)	90	90	Randomization	Coincide	Symptomatic treatment	Symptomatic treatment+TCM	➀➂➄➅
Zeng (31)	35	35	Random number table	Coincide	Conventional treatment	Conventional treatment+TCM	➀➁➃➅
Zhang (60)	30	30	Randomization	Coincide	Symptomatic treatment	Symptomatic treatment+TCM	➀➁
Zhang (61)	28	29	Randomization	Coincide	Conventional treatment	Conventional treatment+TCM	➀➁➂➃➄➅
Zhao (32)	30	30	Random number table	Coincide	Danggui Buxue decoction	TCM	➀➁➃➄➅
Zhou (62)	20	20	Randomization	Coincide	EPO	EPO+TCM	➀➁➄

➀ Hemoglobin; ➁ Clinical efficacy; ➂ Red blood cell count; ➃ KPS score; ➄ TCM syndrome score; ➅ TCM syndrome efficacy; ➆ CD3+; ➇ CD4+; ➈ CD8+; ➉ CD4+/CD8+.

**FIGURE 2:**
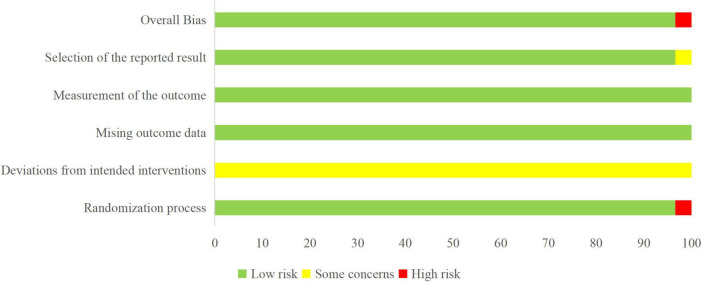
Risk-of-bias graph.

**FIGURE 3:**
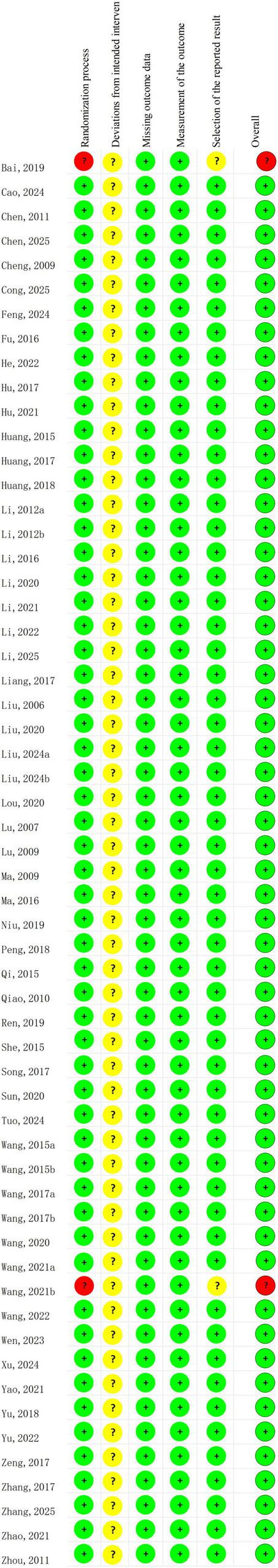
Risk-of-bias summary.

Among the 58 included clinical studies, 27 studies (7–32) employed random number table for group allocation, 2 studies (33, 34) employed the envelope method, 2 studies (11, 35) used a lottery method, and 2 studies (36, 37) applied simple randomization. The remaining 25 studies (9, 35, 38–62) only mentioned randomization without specifying the method. Additionally, allocation concealment was inadequate in 2 studies (36, 56). Blinding was not mentioned in any of the studies. All studies reported complete outcome data with no selective reporting. See Figures 2, 3.

### 3.3 Meta-analysis results

#### 3.3.1 Primary outcome measure

##### 3.3.1.1 Analysis of hemoglobin

**FIGURE 4:**
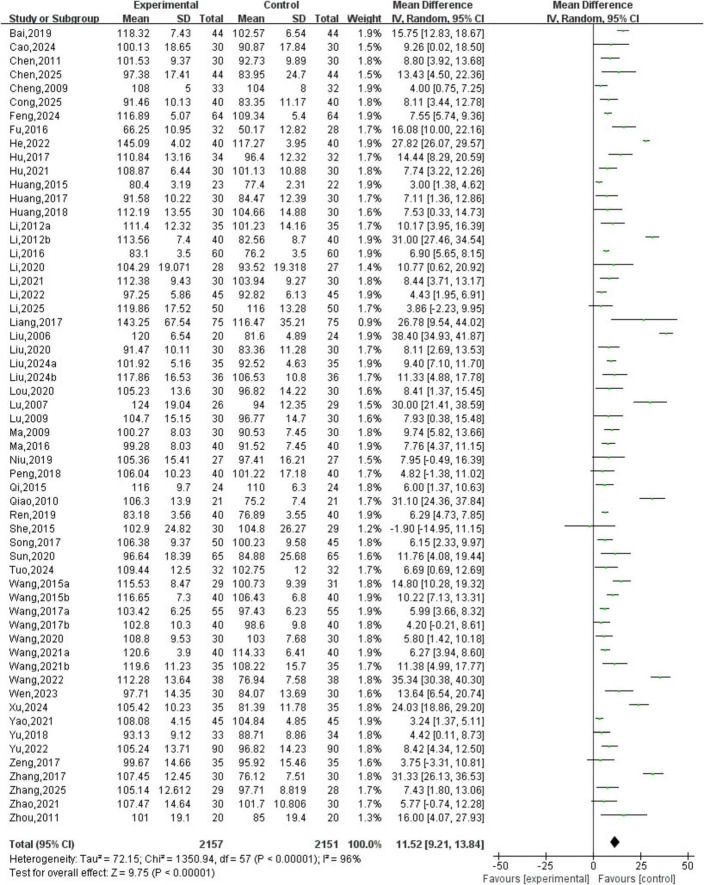
Hemoglobin analysis.

A total of 58 studies involving 4,308 patients were included. The heterogeneity testing indicated significant heterogeneity among the 58 studies (*P* < 0.05, I^2^ = 96%), and thus a random-effects model was applied. The meta-analysis demonstrated a statistically significant difference in hemoglobin levels between the two groups (MD = 11.52, 95% CI [9.21, 13.84], *P* < 0.05). See Figure 4.

##### 3.3.1.2 Publication bias

**FIGURE 5:**
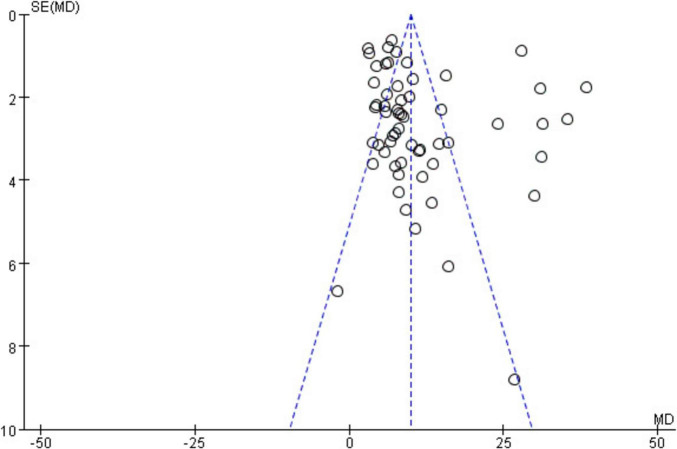
Funnel diagram of hemoglobin analysis.

A funnel plot was constructed for the primary outcome measure. The results demonstrated that the funnel plot was largely symmetrical on both sides, indicating a low likelihood of publication bias in this study. It can be concluded that TCM significantly increases hemoglobin levels and improves anemia in patients. See Figure 5.

##### 3.3.1.3 Subgroup analysis

**FIGURE 6:**
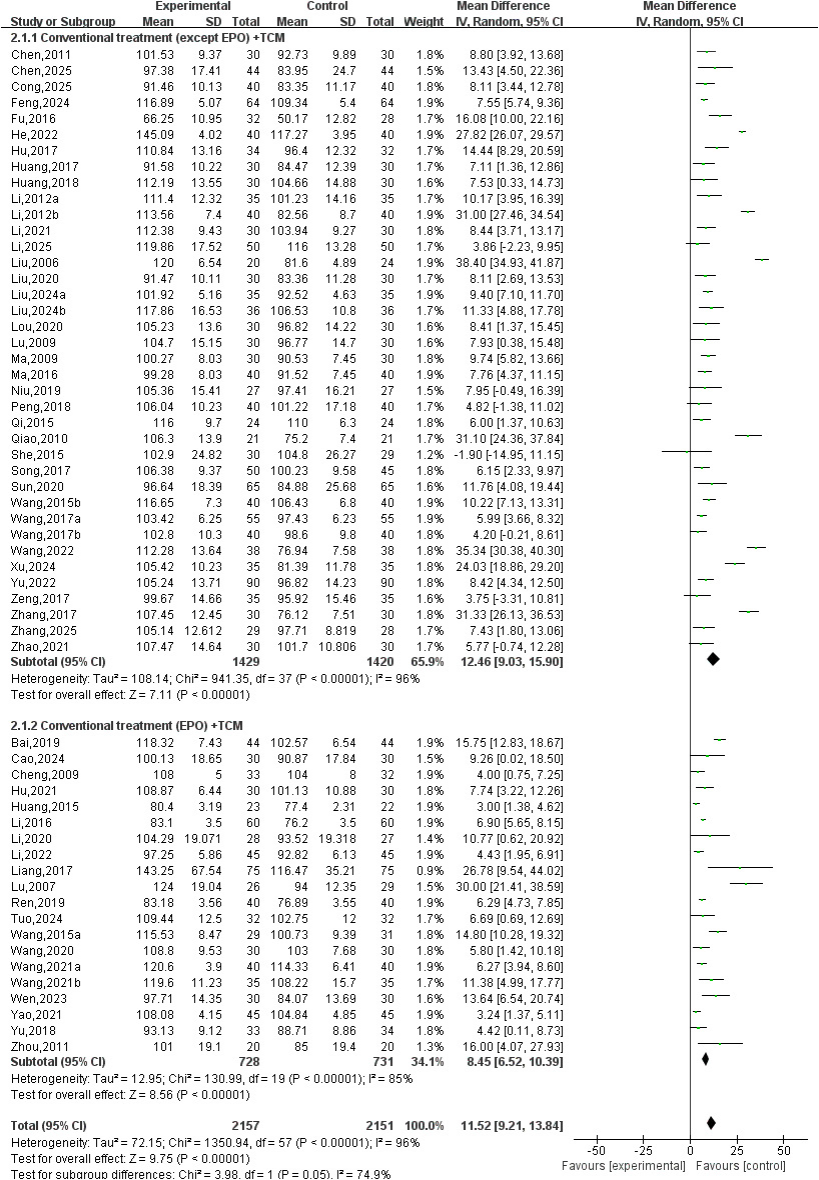
Subgroup analysis of hemoglobin.

A subgroup analysis of hemoglobin levels was performed, with subgroups stratified based on whether conventional treatment involved EPO. The interventions were divided into two subgroups: conventional treatment (excluding EPO) combined with TCM and conventional treatment (including EPO) combined with TCM. The subgroup analysis revealed that the overall results remained consistent regardless of whether EPO was included in the conventional treatment. TCM was identified as the core component of the intervention, suggesting that the study findings are robust. See Figure 6.

#### 3.3.2 Secondary outcome measures

##### 3.3.2.1 Analysis of clinical efficacy

**FIGURE 7:**
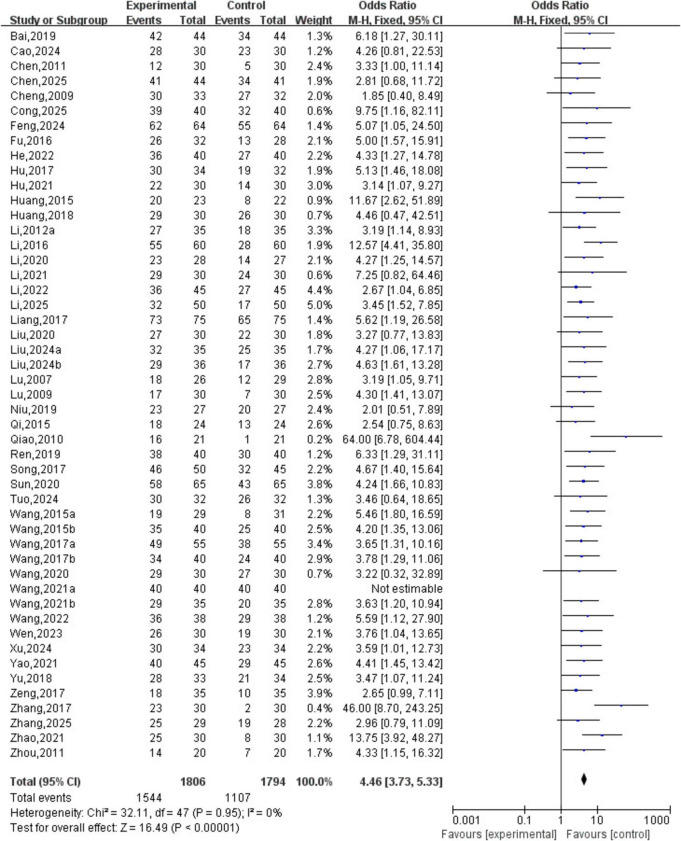
Clinical effect analysis.

A total of 49 studies involving 3,600 patients were included. The heterogeneity testing indicated homogeneity among the 49 studies (*P* > 0.05, I^2^ = 0%), and thus a fixed-effects model was applied. The meta-analysis demonstrated a statistically significant difference in clinical efficacy between the two groups (OR = 4.46, 95% CI [3.73, 5.33], *P* < 0.05). See Figure 7.

##### 3.3.2.2 Analysis of red blood cell count

**FIGURE 8:**
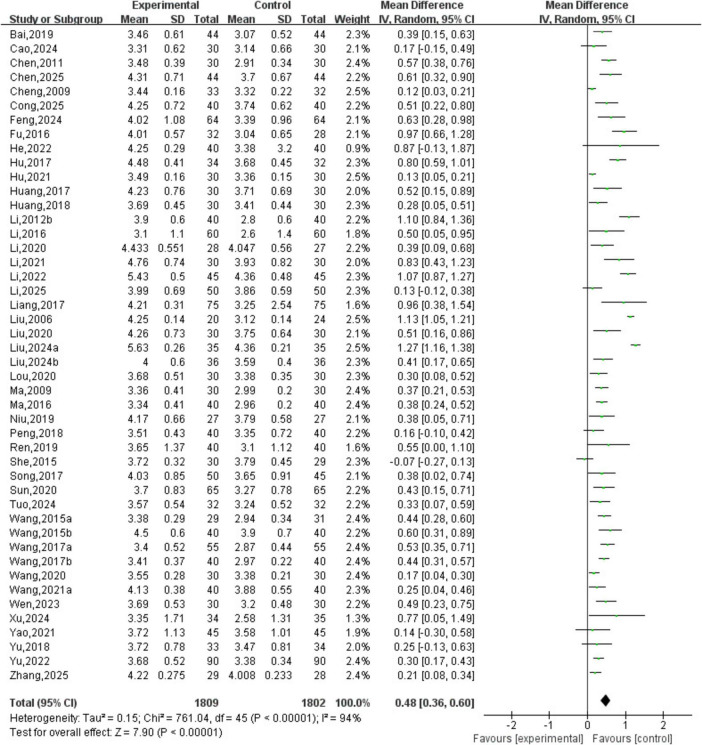
Red blood cell count analysis.

A total of 46 studies involving 3,611 patients were included. The heterogeneity testing indicated significant heterogeneity among the 46 studies (*P* < 0.05, I^2^ = 94%), and thus a random-effects model was applied. The meta-analysis demonstrated a statistically significant difference in red blood cell count between the two groups (MD = 0.48, 95% CI [0.36, 0.60], *P* < 0.05). See Figure 8.

##### 3.3.2.3 Analysis of KPS scores

**FIGURE 9:**
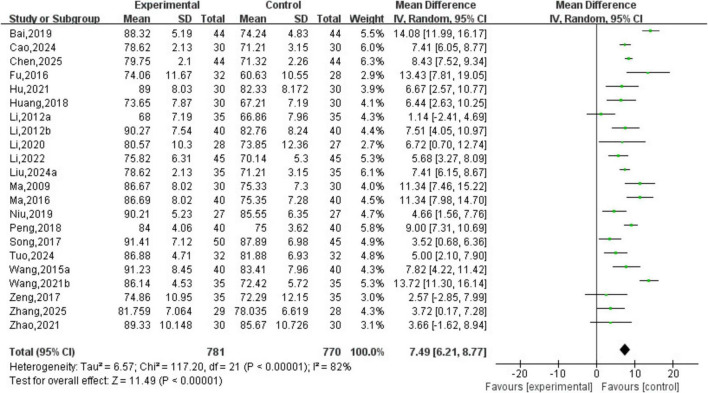
Karnofsky Performance Status (KPS) scores analysis.

A total of 22 studies involving 1,551 patients were included. The heterogeneity testing indicated significant heterogeneity among the 22 studies (*P* < 0.05, I^2^ = 82%), and thus a random-effects model was applied. The meta-analysis demonstrated a statistically significant difference in KPS scores between the two groups (MD = 7.49, 95% CI [6.21, 8.77], *P* < 0.05). See Figure 9.

##### 3.3.2.4 Analysis of TCM syndrome score

**FIGURE 10:**
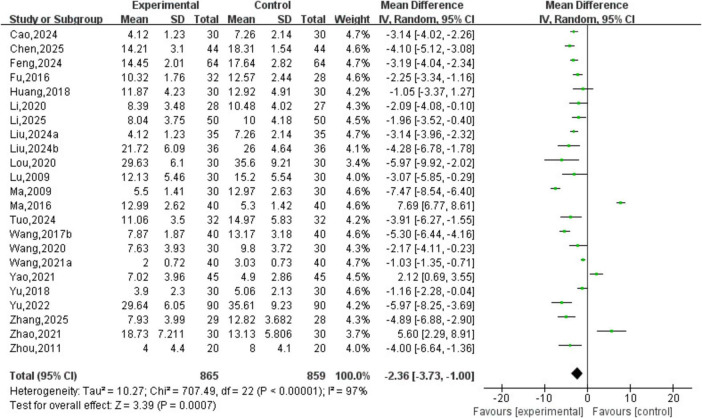
Traditional Chinese Medicine (TCM) syndrome scores analysis.

A total of 23 studies involving 1,724 patients were included. The heterogeneity testing indicated significant heterogeneity among the 23 studies (*P* < 0.05, I^2^ = 97%), and thus a random-effects model was applied. The meta-analysis demonstrated a statistically significant difference in TCM syndrome scores between the two groups (MD = −2.36, 95% CI [−3.73, −1.00], *P* < 0.05). See Figure 10.

##### 3.3.2.5 Analysis of TCM syndrome efficacy

**FIGURE 11:**
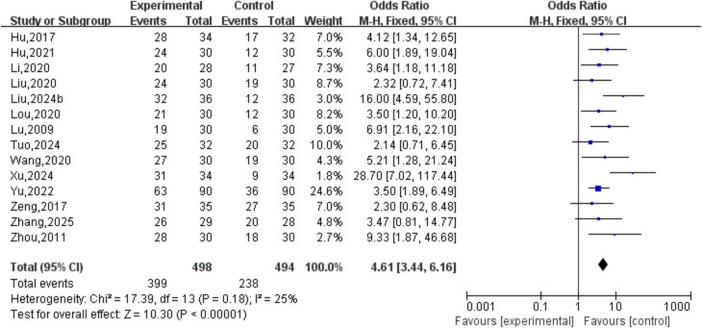
Curative effect analysis of TCM syndromes.

A total of 14 studies involving 992 patients were included. The heterogeneity testing indicated homogeneity among the 10 studies (*P* > 0.05, I^2^ = 0%), and thus a fixed-effects model was applied. The meta-analysis demonstrated a statistically significant difference in TCM syndrome efficacy between the two groups (OR = 4.61, 95% CI [3.44, 6.16], *P* < 0.05). See Figure 11.

##### 3.3.2.6 Immune function analysis

**FIGURE 12:**
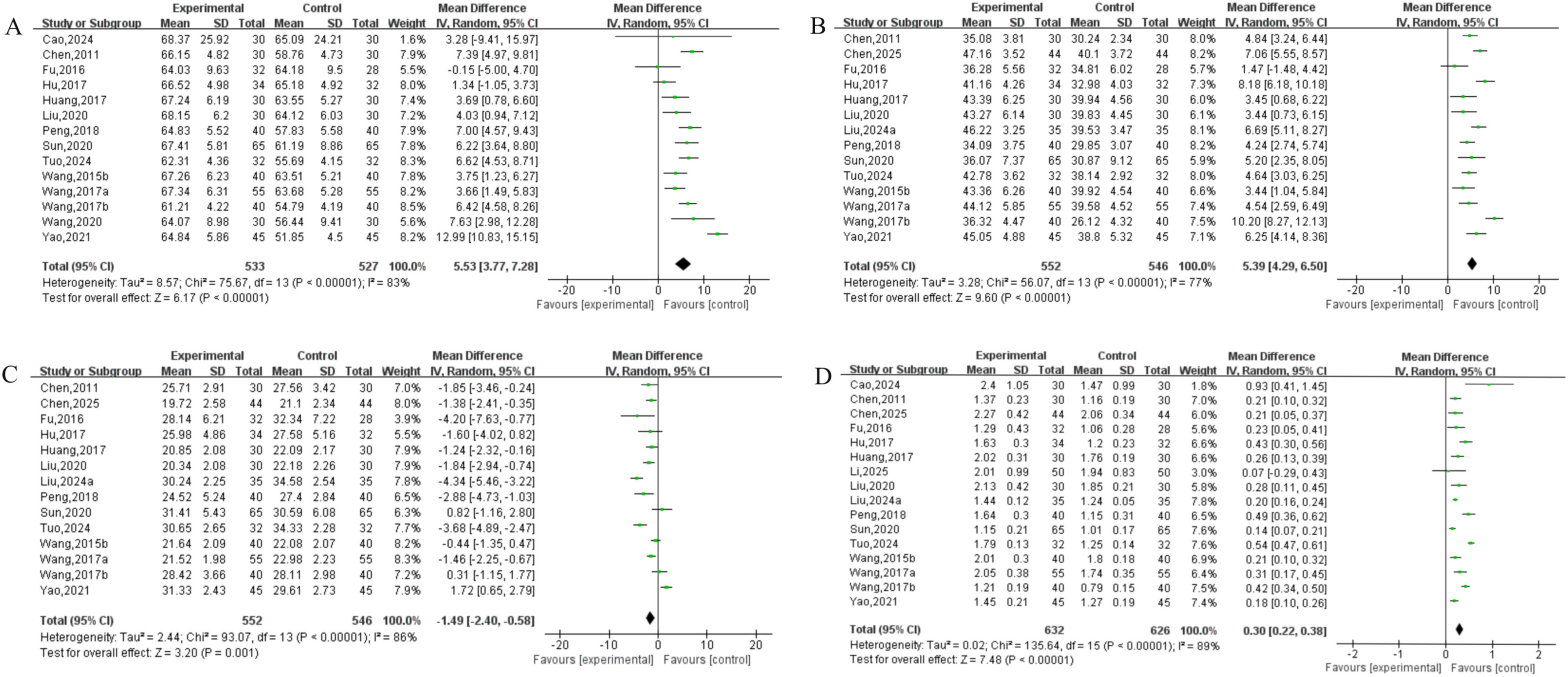
Immune function analysis **(A)** CD3+, **(B)** CD4+, **(C)** CD8+, **(D)** CD4+/CD8+.

Analysis of CD3+ cells included 14 studies involving 1,060 patients. The heterogeneity testing indicated significant heterogeneity among the 14 studies (*P* < 0.05, I^2^ = 83%), and thus a random-effects model was applied. The meta-analysis demonstrated a statistically significant difference in CD3+ levels between the two groups (MD = 5.53, 95% CI [3.77, 7.28], *P* < 0.05). See Figure 12A. Analysis of CD4+ cells included 14 studies involving 1,098 patients. The heterogeneity testing indicated significant heterogeneity among the 14 studies (*P* < 0.05, I^2^ = 77%), and thus a random-effects model was applied. The meta-analysis demonstrated a statistically significant difference in CD4+ levels between the two groups (MD = 5.39, 95% CI [4.29, 6.50], *P* < 0.05). See Figure 12B. Analysis of CD8+ cells included 14 studies involving 1,098 patients. The heterogeneity testing indicated significant heterogeneity among the 14 studies (*P* < 0.05, I^2^ = 86%), and thus a random-effects model was employed. The meta-analysis demonstrated a statistically significant difference in CD8+ levels between the two groups (MD = −1.49, 95% CI [−2.40, −0.58], *P* < 0.05). See Figure 12C. Analysis of the CD4+/CD8+ included 16 studies involving 1,258 patients. The heterogeneity testing indicated significant heterogeneity among the 16 studies (*P* < 0.05, I^2^ = 89%), and thus a random-effects model was applied. The meta-analysis demonstrated a statistically significant difference in the CD4+/CD8+ between the two groups (MD = 0.30, 95% CI [0.22, 0.38], *P* < 0.05). See Figure 12D.

#### 3.3.3 Grading evidence

A total of 10 outcome indicators were systematically evaluated in this study. None of the included studies involved allocation concealment or blinding, which was rated as a serious risk of bias. The literature included for the Hemoglobin and TCM syndrome efficacy outcomes demonstrated homogeneity, therefore, the inconsistency of the remaining outcome indicators was rated as serious. All outcome indicators were considered direct evidence, with no indirectness present. The data from the literature included in this study were complete, with no selective reporting identified. See Table 2.

**TABLE 2:** Grading evidence of the included literature.

Outcome measures	Included literature	Bias risk	Inconsistency	Indirect	Uncertainty	Publication bias	Group		Statistical measure (95% CI)	Grading
							Control group	Treatment group		
Hemoglobin	58	Serious	No	No	No	No	2157	2151	MD = 11.52, 95% CI [9.21, 13.84]	Middle
Clinical efficacy	49	Serious	Serious	No	No	No	1806	1794	OR = 4.46, 95% CI [3.73, 5.33]	Low
Red blood cell count	46	Serious	Serious	No	No	No	1809	1802	MD = 0.48, 95% CI [0.36, 0.60]	Low
KPS score	22	Serious	Serious	No	No	No	781	770	MD = 7.49, 95% CI [6.21, 8.77]	Low
TCM syndrome score	23	Serious	Serious	No	No	No	865	859	MD = −2.36, 95% CI [−3.73, −1.00]	Low
TCM syndrome efficacy	14	Serious	No	No	No	No	498	494	OR = 4.61, 95% CI [3.44, 6.16]	Middle
CD3+	14	Serious	Serious	No	No	No	533	527	MD = 5.53, 95% CI [3.77, 7.28]	Low
CD4+	14	Serious	Serious	No	No	No	552	546	MD = 5.39, 95% CI [4.29, 6.50]	Low
CD8+	14	Serious	Serious	No	No	No	552	546	MD = −1.49, 95% CI [−2.40, −0.58]	Low
CD4+/CD8+	16	Serious	Serious	No	No	No	632	626	MD = 0.30, 95% CI [0.22, 0.38]	Low

Allocation concealment and blinding are not involved (downgraded by one level). Heterogeneity exists in the included literature (downgraded by one level).

## 4 Discussion

Traditional Chinese Medicine has a long history in the treatment of anemia and possesses unique advantages. CRA falls within the categories of “blood depletion” and “consumptive disease” in TCM, primarily characterized by deficiency of qi and blood. Its disease location is in the spleen and kidney, and it is closely related to the liver. According to TCM theory, in patients with malignant tumors, prolonged illness leads to visceral deficiency, or radiotherapy and chemotherapy consume healthy qi, resulting in dysfunction of the spleen and stomach in transportation and transformation, which consequently leads to insufficient generation and transformation of qi and blood. Additionally, patients often present with qi stagnation, blood stasis, phlegm coagulation, dampness accumulation, and heat toxin, which affect the liver’s function of storing blood, leading to a reduction in blood volume. The kidney stores essence, prolonged illness damages the kidney, leading to kidney essence deficiency and liver blood depletion, which consumes blood and injures essence. Under the combined action of “cancer” and “toxin,” the key pathogenesis characterized primarily by “deficiency” accompanied by “toxin and stasis” is formed (63). Therefore, the treatment principle should focus on tonifying deficiency, combined with resolving toxin and removing stasis.

A total of 48 studies on TCM for the treatment of CRA were included in this analysis. The results demonstrated that the combination of TCM with conventional treatment or the use of TCM alone significantly increased hemoglobin (MD = 11.52, 95% CI [9.21, 13.84], *P* < 0.05) and improved clinical efficacy (OR = 4.46, 95% CI [3.73, 5.33], *P* < 0.05) in patients with CRA compared to conventional treatment alone. Improvements were also observed in red blood cell count, KPS score, TCM syndrome score, and TCM syndrome efficacy. Furthermore, TCM treatment exhibited a positive immunomodulatory effect, as indicated by increased proportions of CD3+ and CD4+ T cells and an elevated CD4+/CD8+. Funnel plot analysis of hemoglobin showed approximate symmetry, suggesting a low risk of publication bias and supporting the reliability of the study conclusions. These findings indicate that TCM may provide an effective adjunctive strategy for the comprehensive management of CRA. In addition, some of the included studies compared the safety profiles of TCM and conventional treatment. Overall, conventional treatment for CRA was associated with adverse reactions such as dizziness, headache, abdominal pain, bloating, nausea, vomiting, skin allergies, and even abnormal liver function. In contrast, TCM treatment for CRA not only enhanced clinical efficacy but also resulted in a significantly lower incidence of adverse reactions compared to conventional treatment.

From a modern medical perspective, TCM ameliorates CRA through multi-target mechanisms. The study found that TCM can improve immune function indicators, which is related to its ability to modulate the immunosuppressive state within the tumor microenvironment and mitigate inflammatory responses. Inflammatory responses can lead to anemia by regulating hepcidin, thereby inhibiting iron absorption and release and causing iron metabolism disorders (64). Many TCM herbs for replenishing Qi and nourishing blood possess anti-inflammatory properties, stimulate bone marrow hematopoiesis, and enhance immune function. This aligns with the TCM theory of “fortifying the spleen and tonifying the kidney, replenishing Qi and nourishing blood” for improving CRA and provides further evidential support for the clinical efficacy observations in this study.

The findings of this study are consistent with earlier meta-analyses by Huang et al. (5) and Dang et al. (6), all supporting the positive efficacy of TCM for CRA. However, the study significantly extends and deepens previous meta-analyses in several key aspects. First, the literature search was updated to 2025 and covered multiple international databases, incorporating high-quality clinical studies published in recent years, thereby enhancing the timeliness and comprehensiveness of the evidence. Second, this study strictly adhered to the PRISMA guidelines and, for the first time in a systematic review on this topic, applied the Cochrane RoB 2.0 tool and the GRADE approach to standardize the assessment of evidence quality, making the conclusions of this study more objective and standardized.

## 5 Limitations

There are some limitations in the application of TCM for CRA. Firstly, the protocol for this systematic review was not prospectively registered, and the number of cases in the literature included in this study was relatively small, and large-sample clinical studies are lacking. Moreover, there is no standardized protocol for treatment duration and observation periods in clinical study. Secondly, variations in participant characteristics across the included studies resulted in significant clinical heterogeneity, which may affect the assessment of therapeutic efficacy. Finally, most studies had a relatively high risk of bias, and two studies included in this study did not adequately implement blinding, which may introduce selection bias, performance bias, and measurement bias (65). Therefore, future large-sample, multicenter, prospective studies are warranted to evaluate the clinical efficacy of TCM in treating CRA, with the aim of providing higher-quality medical evidence.

## 6 Conclusion

In summary, current clinical evidence indicates that as an effective complementary therapy for CRA, TCM can safely and effectively improve hemoglobin, immune function, clinical symptoms, and quality of life in patients with CRA. In clinical practice, for CRA patients who respond poorly to conventional treatments or cannot tolerate its side effects, considering the combined use of TCM under the guidance of TCM syndrome differentiation by a qualified TCM practitioner represents a reasonable therapeutic option. However, given the overall low certainty of the current evidence, it is presently insufficient to formulate a strong recommendation for the routine inclusion of TCM in CRA treatment guidelines. The primary value of this study lies in systematically reviewing the existing evidence, clarifying its strengths and limitations, and providing direction for future pivotal research aimed at generating higher-level evidence.

## Data Availability

The original contributions presented in this study are included in this article/supplementary material, further inquiries can be directed to the corresponding author.
